# Incremental Material Flow Analysis with Bayesian Inference

**DOI:** 10.1111/jiec.12698

**Published:** 2017-11-27

**Authors:** Richard C. Lupton, Julian M. Allwood

**Affiliations:** Department of Engineering, University of Cambridge, Trumpington St., CB2 1PZ Cambridge, United Kingdom

**Keywords:** Bayesian inference, industrial ecology, Markov Chain Monte Carlo, material flow analysis, steel, uncertainty

## Abstract

Material flow analysis (MFA) is widely used to study the life cycles of materials from production, through use, to reuse, recycling, or disposal, in order to identify environmental impacts and opportunities to address them. However, development of this type of analysis is often constrained by limited data, which may be uncertain, contradictory, missing, or over‐aggregated.

This article proposes a Bayesian approach, in which uncertain knowledge about material flows is described by probability distributions. If little data is initially available, the model predictions will be rather vague. As new data is acquired, it is systematically incorporated to reduce the level of uncertainty.

After reviewing previous approaches to uncertainty in MFA, the Bayesian approach is introduced, and a general recipe for its application to material flow analysis is developed. This is applied to map the global production of steel using Markov Chain Monte Carlo simulations. As well as aiding the analyst, who can get started in the face of incomplete data, this incremental approach to MFA also supports efforts to improve communication of results by transparently accounting for uncertainty throughout.

**Supplementary Information:**

The online version of this article (doi:10.1111/jiec.12698) contains supplementary material, which is available to authorized users.

## Introduction

The threat of climate change has led to global agreement (at COP21 Paris, in 2015) on the need to limit mean temperature rise to 2 °C or less. Ensuring an acceptable probability of keeping within this limit will require much more dramatic cuts in emissions in industrialized countries than have been achieved to date (Anderson and Bows 2011). As approximately one‐third of industrial and energy emissions are associated with making materials and goods, and as there are limited options to reduce these emissions using upstream measures (Allwood and Cullen 2012), it is crucial to understand the whole life cycle of materials from production, through use, to reuse, recycling, or disposal, in order to identify opportunities to improve the efficiency with which embodied‐emissions‐intensive materials are used.

Material flow analysis (MFA) is widely used to understand these material systems and the associated environmental impacts, whether carbon emissions associated with steelmaking (Milford et al. 2013) or releases of nanoparticles to the environment (Gottschalk et al. 2010). It can be applied at the level of whole economies (Fischer‐Kowalski et al. 2011), regionally (Baccini and Brunner 2012), or for particular sectors (Cullen et al. 2012); it can be used to give a static snapshot of a single year or to show accumulations of stock over time in dynamic MFA (Müller et al. 2004). MFA describes material systems in terms of *flows* of materials moving between *processes* (Brunner and Rechberger 2003), which may represent physical transformation processes (such as blast furnaces), societal subsystems (such as households), or ecological compartments (such as rivers).

In this type of analysis data limitations are inevitable, whether from inaccuracies in sampling or measurement, extrapolation of data collected for different regions, products, or time periods, or because data is simply not available. This leads to “epistemic” uncertainty (due to lack of knowledge), on top of the “aleatory” uncertainty due to inherent variability in the system (Laner et al. 2014). Dealing with this uncertainty is important for two reasons. Firstly, it helps with the interpretation of the results of the analysis, by showing which numbers or comparisons are significant (Danius and Burström 2009); conversely, ignoring uncertainty raises doubts about the reliability of results (Laner et al. 2014). Secondly, by acknowledging that developing an MFA is an iterative, incremental process (Brunner and Rechberger 2003), tracking uncertainty helps the analyst. First iterations can begin even without good data, producing results with high uncertainty, which can be reduced as more data becomes available. At each iteration, knowledge of uncertainties allows prioritization of data collection and modeling effort.

Laner and colleagues (2014) reviewed the methods used to deal with uncertainty in MFA studies and identified three types of approaches. In the first, *qualitative descriptions* of confidence in results are chosen by the analyst, such as “high confidence” or “very low confidence” (e.g. Graedel et al. 2004). In the second type, more *formal methods for assigning confidence* are used (Hedbrant and Sörme 2001; Laner et al. 2015), based on information such as the source of the data and how specific it is, the transparency of its provenance, or other means of characterizing the data (Schwab et al. 2017). In the third type of approach, *quantitative statistical methods* are used to describe uncertainty. The first two types of approach tackle the issue of communicating uncertainty of data and results, but do not provide a framework to track, update, and propagate uncertainty, leaving the burden on the analyst to judge this themselves. To achieve the vision above of incremental development of MFA using uncertainty, the third type of approach, quantitative statistical methods, are needed.

**Figure 1:**
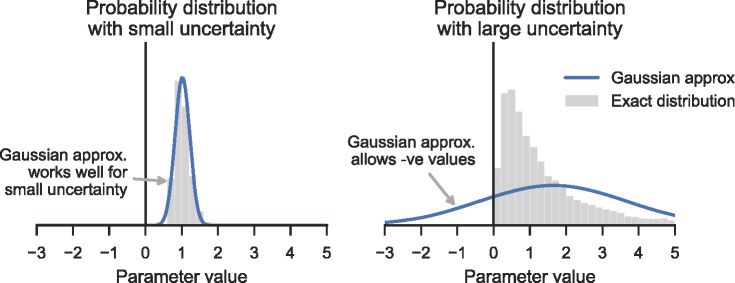
The Gaussian approximation works well when uncertainties are small and symmetric (left). The approximation can be poor for large uncertainties that occur during early iterations of an MFA, such as when a quantity must be positive but its value is poorly known (right). In this case the Gaussian approximation describes a significant chance that the value is negative, which is physically meaningless.

The statistical methods identified by Laner and colleagues (2014) are of four types:

1. *Sensitivity analysis* considers possible variations in model parameters, but does not necessarily seek to track actual knowledge about uncertainty in the parameters. For example, Milford and colleagues (2013) considered ± 10% variations in the parameters controlling their model of carbon emissions associated with steelmaking, but these were intended to give insight into the behavior of the model and are not meant to imply that each parameter value is known to that level of certainty.
2. Methods based on *Gaussian error propagation*, sometimes including data reconciliation, quantify uncertainty in parameters using standard deviations, and propagate this uncertainty through the model to find the standard deviation of results. For example, Bader and colleagues (2011) used this method in analyzing stocks of copper in Switzerland. This can be very effective when the Gaussian approximation is appropriate, but it can break down when uncertainties are large (figure 1) making it unsuitable for tracking uncertainty from the early iterations of an analysis.
3. “*Mathematical MFA*” approaches (Baccini and Bader 1996) relax the assumption of Gaussian distributions, instead using arbitrary probability distributions to represent uncertainty, which are propagated through the model using Monte Carlo simulations. For example, Glöser and colleagues (2013) used uniformly‐distributed parameters to assess uncertainty in recycling indicators for copper, while Gottschalk and colleagues (2010) used uniform, lognormal, and triangular distributions of parameters to find concentrations of nanoparticles in the environment, using a method they call “probabilistic MFA”. Later, Bornhöft and colleagues (2016) extended this method to include dynamic MFA models. These methods see the process of MFA development as iterative. Sensitivity analysis has been used to prioritize areas which need improvement in the next iteration (Do‐Thu et al. 2011) but formal methods for tracking and updating knowledge about uncertain model parameters are not typically used.
4. Methods based on *possibility theory* use fuzzy intervals to represent uncertainty. This allows information to be specified in a less precise way than by using probability distributions, which may be appropriate when only vague information is known about the system. Dubois and colleagues (2014) present an approach using fuzzy intervals for data reconciliation in linear MFA models. Džubur and colleagues (2017) describe a reconciliation algorithm which can handle more general non‐linear membership functions. They use the “degree of consistency” between data sources and the model to assess data quality and drive iterative improvements to model or data.

Of these, the probabilistic and possibilistic approaches are therefore most suitable for describing uncertainty through the development of a material flow analysis. A method of tracking and updating knowledge about the model is needed to support an incremental approach. In other fields, Bayesian inference (Jaynes and Bretthorst 2003) has provided a successful framework for systematic understanding of uncertain knowledge, especially in recent years as more problems have become computationally feasible. In this framework, the analyst's current state of knowledge about the model is tracked using probability distributions. If little data is initially available, the initial model predictions will be rather vague, but as more data is acquired it can be incorporated in a rigorous way to reduce the level of uncertainty.

The Bayesian inference approach has been discussed in connection with MFA by Gottschalk and colleagues (2010), who mentioned inference as an extension of probabilistic MFA, but as there was no real data available in their study they did not develop the idea further. Cencic and Frühwirth (2015) used a Bayesian approach for data reconciliation, for the case that the available data relates directly to flows in the model, and the model is expressed as linear constraints. However, there is a large class of useful data which are not direct observations of flows (e.g., aggregated observations of many flows, or knowledge about percentage inputs/outputs of a process), and in many cases models cannot be expressed as linear constraints, limiting the applicability of that method. This article builds on these to develop a Bayesian inference approach to all types of MFA, allowing knowledge about flow rates, stock accumulations and other parameters to be incrementally built up as new data becomes available.

Bayesian inference is a very general framework, so the following section examines what needs to be done to apply it to a new area, resulting in a list of requirements that must be met to use an MFA model in the Bayesian inference framework. Then, a case study of a static Bayesian MFA model is presented, which is used to incrementally map global steel flows from incomplete and uncertain data, before the last section concludes with a discussion of results and future developments.

## What Is Required to Apply Bayesian Inference to MFA?

**Figure 2:**
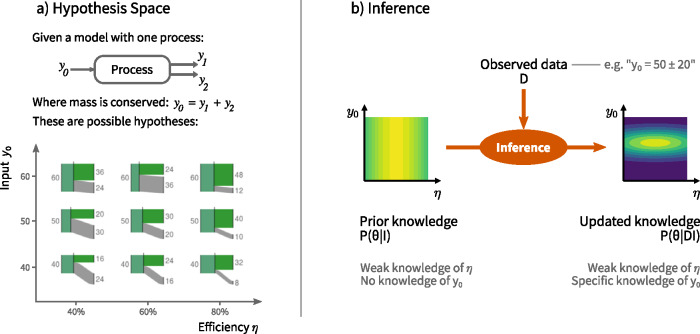
Material flow analysis in a Bayesian inference framework. (a) For a simple one‐process MFA model, there are a range of possible hypotheses consistent with the mass‐balance constraints and other assumptions. The small Sankey diagrams illustrate 9 possibilities drawn from the hypothesis space, arranged according to two model parameters, the input y_0_ and the efficiency η. (b) The inference process takes new observed data and combines it with the predictions of the model to update knowledge about the model parameters. In this sketch, a measurement of y_0_ results in knowledge about parameters becoming more specific (i.e., the probability distribution becomes more peaked).

The development of an MFA analysis typically starts with the choice of system boundaries, and definition of the processes and stocks to be considered as well as the flows of goods and substances which connect them, to yield an idealized mathematical model of the system being studied (Brunner and Rechberger 2003). The model includes equations enforcing conservation of mass, and may include further relationships describing the behavior of processes within the system. By setting up these governing equations, the modeler is setting the scope of the possible situations to be considered in the analysis. Together these form the “hypothesis space”, where each hypothesis is a particular possible (although not necessarily likely) configuration of the system, each consistent with the model equations. For example, figure 2a shows a simple MFA model with one process, with a single governing equation ${y}_0={y}_1+{y}_2$ (conservation of mass), and the corresponding hypothesis space. Traditionally, the next step is *calibration*, in which data is collected and used to pick out the one “correct” or best‐estimate hypothesis. In contrast, in the Bayesian approach, probability distributions are used to track knowledge about the relative merit of all the hypotheses, giving a more nuanced picture of what is known about the system.

Rather than explicitly listing individual hypotheses, such as the nine Sankey diagrams shown in figure 2a, generally it is more convenient to work with a continuous space of hypotheses. To do this, a set of *model parameters*, denoted θ, are introduced, whose purpose is to uniquely define a particular configuration of the model. To describe the example one‐process model uniquely, two parameters are needed; in figure 2a, the input *y*_0_ and the efficiency η are used, from which the output flows can be found as ${y}_1=\eta {y}_0$ and ${y}_2={y}_0-{y}_1$ (the mass‐balance constraint). For example, $\left({y}_0=40,\eta =0.4\right)$ refers to the bottom‐left Sankey diagram in figure 2a. The choice of model parameters is not fixed; other choices of parameter, such as $\left({y}_1,{y}_2\right)$ or $\left({y}_0,{y}_1\right)$ also uniquely describe the configuration of the model, and are valid choices; the decision of which to use is mostly a matter of convenience. More generally, realistic MFA models will have a larger hypothesis space defined by more than two model parameters. For a static MFA, possible parameters would be the import flows entering the system, together with the pattern of relative allocation of flows to subsequent processes within the system (e.g., Gottschalk et al. 2010). A dynamic analysis would require additional parameters such as lifetimes of in‐use stocks (e.g., Müller et al. 2004).

When new evidence is acquired, the probability distributions are updated accordingly (figure 2b). This new evidence, denoted generically as the “observed data” *D*, can include data directly observed by the analyst, or collected from other sources. In figure 2b, the updated knowledge $P\left(\theta | DI\right)$ differs from the initial knowledge $P\left(\theta |I\right)$ by being conditional on *D*: it is the probability of a particular set of model parameters (and hence a particular hypothesis), *given that* the data *D* has been observed.^1^ Probabilities in Bayesian inference are conventionally conditional on *I* (Jaynes and Bretthorst 2003), which represents the “background information”: the assumptions and experience which goes into choosing a particular system boundary, model equations, and so on.

This updated state of knowledge about the model parameters, having seen the observed data *D*, can be found from Bayes' theorem:

1 $$ P\left(\theta | DI\right)=\frac{P\left(D|\theta I\right)P\left(\theta |I\right)}{P\left(D|I\right)} $$

The terms appearing in this equation are defined in table Table 1. To apply Bayesian inference to a particular MFA model, the form of each of these probabilities must be determined. In the following, each is considered in turn.

**Table 1:** Steps of Bayesian inference and the corresponding terms in Bayes' theorem

Name	Term in eq 1	Meaning
Likelihood	$P\left(D|\theta I\right)$	How likely is the model to predict the observed data *D* for a specific set of parameter values θ?
Prior	$P\left(\theta |I\right)$	Initial knowledge about parameter values, before seeing *D*
Posterior	$P\left(\theta | DI\right)$	Updated knowledge about parameter values, having seen *D*
Evidence	$P\left(D|I\right)$	Acts as a normalization constant, because *D* is considered fixed (only important when choosing between multiple models)

### Likelihood: Models and Observation

The likelihood $P\left(D|\theta I\right)$ describes the probability that the model would have predicted the actually‐observed data *D*, for particular values of the parameters θ. Due to measurement error, and mismatches between the idealized model and the observed data, the model prediction is not expected to exactly match the observed value. For example, in figure 2a, imagine the parameter values are $\theta =\left({y}_0,\eta \right)=\left(40,0.4\right)$, corresponding to a predicted value for *y*_1_ of ${y}_0\eta =16$. If these were the correct parameter values, the probability of observing ${y}_1=14$ might be quite high, whereas observing ${y}_1=100$ would be very unlikely. So the likelihood depends on both the model equations and the process of observation/measurement itself.

Models can be deterministic or random (Tarantola 2005). In deterministic models, the modeled relationship is assumed to be certain, so the only uncertainty is about the correct parameter values. Most MFA models are deterministic, and so this article focuses on deterministic models, for which the model predictions can be written as an explicit function of the parameters:

2 $$ y=f\left(\theta \right) $$

The second step is the process of observation. The model predictions *y* are not observed directly, but instead some functions ${g}_i(y)$ are observed. For example, the aggregated total of several flows, predicted individually by the model, may be known. The individual values ${d}_i$, which make up the observed data *D*, are measured with some error ${e}_i$:

3 $$ {d}_i={g}_i(y)+{e}_i $$

The particular form of ${g}_i(y)$ and the errors ${e}_i$ must be defined for the particular MFA model and type of observed data. In many cases, the assumption of Gaussian errors is appropriate (Jaynes and Bretthorst 2003). If all the observations are independent, they can be multiplied to give the complete likelihood:

4 $$ P\left(D|\theta I\right)=\prod \limits_iP\left({d}_i|\theta I\right) $$

For the example of figure 2a, the model predictions $y=\left({y}_0,{y}_1,{y}_2\right)$ are given by

5 $$ f\left(\theta \right)=\left[\begin{array}{c}{y}_0\\ {}{y}_0\eta \\ {}{y}_0\left(1-\eta \right)\end{array}\right] $$

where $\theta =\left({y}_0,\eta \right)$. Suppose that the flow *y*_1_ is measured to have a value of *d*_1_ with measurement uncertainty σ_1_, expressed probabilistically as

6 $$ {d}_1\mid {y}_1I\sim \mathcal{N}\left({y}_1,{\sigma}_1\right) $$

where $x\mid A\sim \mathcal{N}\left(\mu, \sigma \right)$ means that, given *A*, *x* is normally distributed with mean μ and standard deviation σ. Using the model equations 5 to substitute ${y}_1={y}_0\eta$, this can be written in terms of the standard normal probability density function as

7 $$ P\left({d}_1|\theta I\right)=\frac{1}{\sigma_1\sqrt{2\pi }}\exp \left(-\frac{{\left({d}_1-{y}_0\eta \right)}^2}{2{\sigma}_1^2}\right) $$

which has a peak at ${d}_1={y}_0\eta$. Further measurements can be defined in the same way. For example, if the flow *y*_0_ was also measured as ${d}_2\pm {\sigma}_2$, this would give

8 $$ P\left({d}_2|\theta I\right)=\frac{1}{\sigma_2\sqrt{2\pi }}\exp \left(-\frac{{\left({d}_2-{y}_0\right)}^2}{2{\sigma}_2^2}\right) $$

In most cases, nothing is known to suggest that this measurement is not independent of the measurement of *d*_1_ above, so the likelihood would be found by substituting equations 7 and 8 into equation 4. If more specific information is available to determine how the measurement of *d*_1_ and *d*_2_ is dependent, the joint distribution $P\left(D|\theta I\right)$ must be defined directly.

### Specific or Vague Initial Knowledge

Initial knowledge about model parameters is described by the prior probabilities $P\left(\theta |I\right)$. In some cases this knowledge may be quite specific, such as “the process efficiency is between 75% and 85%” or “the flow rate is estimated as 130 tonnes per year with a standard deviation of 10 tonnes per year”. In these cases, prior probabilities can be directly assigned using suitable distributions, such as normal, lognormal, or uniform distributions. In other cases, however, initial knowledge about a model parameter may be more vague, such as “the process efficiency is between 0% and 100%” or “the flow rate is positive”. Then a “vague” prior distribution should be assigned, which should reflect the little that is known, but avoid inadvertently implying any further knowledge which could bias the results (Jaynes and Bretthorst 2003).

The choice of prior distribution can be difficult, especially when initial knowledge is vague, and there has been considerable philosophical debate about what a “vague” probability distribution might mean (Kass and Wasserman 1996). More widely, possibility theory (e.g., Dubois et al. 2014) offers one way of addressing this. In modern applied Bayesian inference, a more pragmatic approach is preferred (e.g., by Gelman et al. 2013) in which a convenient prior distribution is assumed, and the effect of different assumptions on the results can be checked through a sensitivity analysis, as with any other assumption. To illustrate this, the following examples are given of possible prior distributions for two common parameter types: flow rates (or other positive quantities) and transfer coefficients (which sum to one).

#### Priors for Flow Rates

A flow rate is a parameter such as “global production of pig iron,” which must have a positive value. If nothing else is known, what prior distribution should be used?

An intuitive response might be to use a uniform distribution $p\left(\theta \right)=\mathrm{const}$, from 0 to ∞ (or some sufficiently‐large number). An alternative is to work on a log scale and set $p\left(\log \theta \right)=\mathrm{const}$, equivalent to the Jeffreys prior $p\left(\theta \right)=1/\theta$, which is scale‐invariant (Jaynes and Bretthorst 2003). These two alternatives represent two plausible descriptions of the parameter: the former assigns equal probability to equal ranges (e.g., 10–20 kilograms [kg] and 90–100 kg), while the latter assigns equal probability to different orders of magnitude (e.g., 1–10 kg and 10–100 kg), equivalent to saying that units of kilograms and tonnes are equally likely to be appropriate.

The difference is only significant for large uncertainties that span multiple orders of magnitude. Even then, if high quality data is available then that will dominate the results, regardless of the choice of prior. If in doubt, different types of vague prior can be tested, and if the model results are sensitive to the choice, then more data may be needed to reach a conclusion.

If, on the other hand, an approximate value and uncertainty range are known, this type of parameter is most commonly represented by a truncated normal or a lognormal distribution (see, e.g., work by Gottschalk et al. 2010).

#### Priors for Transfer Coefficients

Transfer coefficients describe the fractions of a process's output that flow to different destinations. For a process with *N* outputs, there are *N* transfer coefficients ${\alpha}_i$ which must satisfy the constraints ${\alpha}_i>0$ and $\sum \limits_i{\alpha}_i=1$. If this is all that is known about the process outputs, what initial probability distribution for the ${\alpha}_i$ should be chosen, which is as vague as possible to avoid biasing the results?

**Figure 3:**
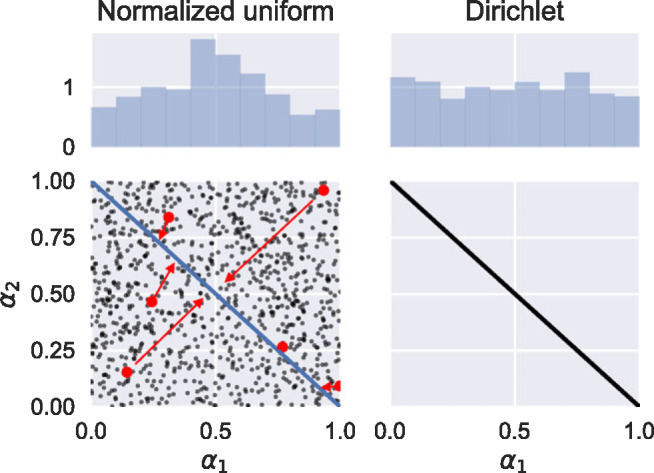
Sampling transfer coefficients uniformly in the range [0, 1], then normalizing, fails to give a uniform distribution (left): the prior is biased towards a 50–50% split. The uniform Dirichlet distribution (right) correctly gives a uniform prior. This figure shows only two transfer coefficients, for ease of plotting, but the same conclusions apply to cases with more coefficients.

One method that has been used in other studies (e.g., Bornhöft et al. 2016) is to sample *N* numbers ${\beta}_i$ from a uniform distribution between 0 and 1, then normalize to give ${\alpha}_i={\beta}_i/\sum \limits_j{\beta}_j$. Although this seems intuitively reasonable, it turns out to produce transfer coefficients that are biased towards allocating equal fractions to each destination (figure 3, left), which is probably not what was intended. A better alternative is to use a uniform Dirichlet distribution (figure 3, right).

If specific information is known about the average values of the transfer coefficients, a concentrated Dirichlet distribution can be used. More complicated distributions that account for correlations between coefficients can be produced from transformed multivariate Gaussian distributions (Gelman et al. 1996; Pawlowsky‐Glahn et al. 2015).

#### Example

**Figure 4:**
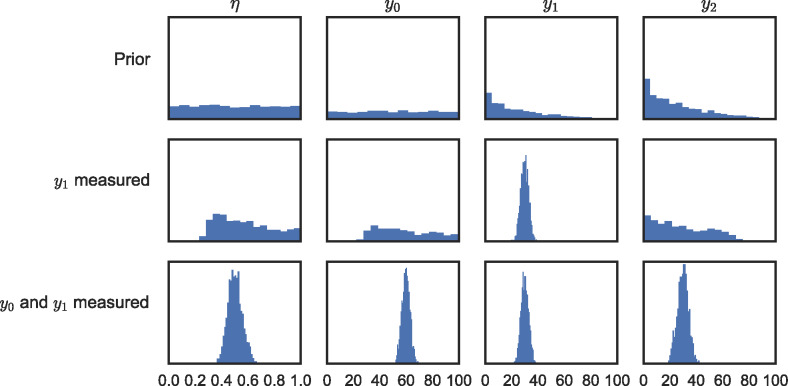
Prior (top row) and posterior (middle & bottom rows) distributions for the one‐process model shown in figure 2a.

For the example model shown in figure 2a, suppose that nothing is known about the process efficiency, while it is known that the process input is less than 100. Prior distributions are chosen to reflect this initial knowledge: the efficiency η is assigned a uniform prior between 0 and 1, and the process input *y*_0_ is assigned a uniform prior between 0 and 100. These distributions are shown in the top row of figure 4, along with the consequential distributions for *y*_1_ and *y*_2_ according to the model equations 5.

### Inference: Updating Knowledge about Parameters from Observed Data

Once vague or specific initial knowledge is defined as prior distributions, and the model and likelihood are defined, Bayes' theorem (equation 1) can be used to find the posterior distribution, which describes the improved knowledge about model parameters as a result of the new, observed data. Although in some cases it is possible to find analytical results, generally the posterior distribution is found approximately using numerical stochastic methods.

To get an intuitive understanding of the procedure, imagine taking every possible hypothesis (i.e., every combination of parameter values), and calculating the model predictions. For each, find the prior probability of the hypothesis, and the likelihood that the model predictions are consistent with the observed data (as discussed above). Combining these according to equation 1 gives the posterior probability of that hypothesis. If each hypothesis is kept or rejected with a probability proportional to the posterior probability, the kept hypotheses form a set of samples which are representative of the likely parameter values, taking into account both the initial knowledge and the observed data.

In practice, for any realistic number of parameters, the number of possible hypotheses is extremely large and it is not possible to test them all. Markov Chain Monte Carlo sampling is a technique for randomly exploring the possible parameter combinations, focusing on the most interesting (most likely) hypotheses (Gelman et al. 2013, chapter 10). The result is a set of samples which are statistically representative of the current knowledge about the model. These can be used to calculate best‐estimate values by averaging, to visualize variation through histograms, to find probabilities of certain events occurring, or directly visualized as Sankey diagrams to show individual possible situations.

There are various algorithms for carrying out the sampling. Classic ones such as Metropolis‐Hastings (Gelman et al. 2013, chapter 11) are simple but need tuning to work efficiently, requiring a more detailed understanding from the analyst. More recent algorithms such as the No U‐Turn Sampler (Hoffman and Gelman 2014) and ensemble samplers (Goodman and Weare 2010) require less tuning to the point that they can be fairly easily used as a routine tool. In this article, the No U‐Turn Sampler implemented in PyMC3 (Salvatier et al. 2016) was used.

#### Example

Continuing the example from the previous section, posterior distributions were found representing updated knowledge about the model parameters in two stages: after measuring ${y}_1=30\pm 3$ (equation 7) and after measuring ${y}_0=60\pm 3$ (equation 8). The resulting distributions are shown in the middle and bottom rows of figure 4.

After the first measurement, the value of *y*_1_ is well known, but the remaining parameters are still only vaguely known (figure 4, middle row). This is because there are multiple situations which could lead to the observed data: the throughput could be high, with a low efficiency, or it could be lower, with a high efficiency. Nonetheless, the distributions have adjusted to show that the input *y*_0_ cannot be less than the output *y*_1_, and the efficiency η cannot be less than about 0.3 (due to the maximum value of *y*_0_ being 100).

After the second measurement, all parameters and outputs are known to within the measurement uncertainty (figure 4, bottom row).

### Summary

To apply Bayesian inference to material flow analysis, the following should be done:

1. The particular MFA model should be formulated as a deterministic model, which predicts outputs (such as flow rates and stock levels) based on a set of parameters. This encompasses the use of most, if not all, existing MFA modeling approaches.
2. The relationship between model predictions and observed data should be defined, including any aggregation, and the confidence in the observed value.
3. Initial knowledge about model parameters should be quantified as probability distributions.

This is a recipe for applying Bayesian inference to MFA. In the following section, a concrete example is given for a particular MFA model, applied to map global flows of steel.

## Case Study: Global Steel Flows

The Bayesian inference approach discussed above is now tested by mapping global steel flows, following Cullen and colleagues (2012). The aim is to demonstrate an incremental development of the analysis, by starting with only a limited amount of data. Further data will then be added to iteratively reduce the uncertainty in the results. At every stage, the results should include the values found by Cullen and colleagues as a possibility.

The next three subsections explain the specific model, the observed data, and the initial knowledge applied in the case study, corresponding to the three general requirements identified above. The last subsection then presents the results.

### Model Structure

**Figure 5:**
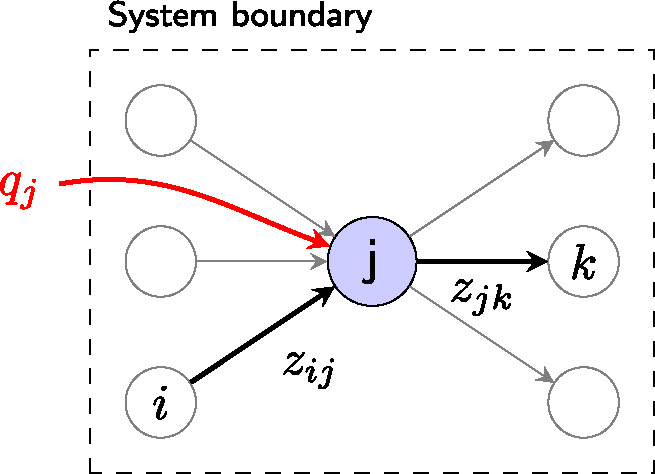
Process units, similar to the one shown in figure 2a, can be assembled to define more complicated model structures.

For the purposes of this case study, a relatively simple model structure is sufficient, as no dynamic stock accumulations are included. The model is built up from a set of connected process units, as shown in figure 5. Conservation of mass determines flows between process units in the model, with the overall pattern of flows being driven by a few external inflows, which in this case include inputs of iron ore and end‐of‐life scrap. Applying conservation of mass at process *j* gives:

9 $$ {q}_j+\sum \limits_i{z}_{ij}=\sum \limits_k{z}_{jk} $$

where ${z}_{jk}$ represents the flow from process *j* to process *k*, and ${q}_j$ is the external inflow to process *j* (figure 5). By introducing transfer coefficients ${\alpha}_{jk}$, which describe the flows leaving process *j* as a fraction of the total output ${x}_j$,

10 $$ {z}_{jk}={x}_j{\alpha}_{jk}\kern8.00em {x}_j=\sum \limits_k{z}_{jk} $$

the conservation equations 9 can be rewritten as

11 $$ {q}_j+\sum \limits_i{x}_i{\alpha}_{ij}={x}_j $$

These mass balances can be assembled into a linear system of equations (as used by, e.g., Gottschalk et al. 2010) which can be solved to give the total flow through each process ${x}_j$:

12 $$ \boldsymbol{x}={\left(\boldsymbol{I}-\boldsymbol{A}\right)}^{-1}\boldsymbol{q} $$

where ${\boldsymbol{A}}_{ij}={\alpha}_{ji}$ and ***I*** is the identity matrix.

The state of the model is therefore characterized by the external inflows ***q*** and the transfer coefficient matrix ***A***, but it is inconvenient to work with this matrix directly as it contains many zeros, and its columns must sum to 1. Instead, each column of the matrix is defined by a sub‐model for each process unit:

13 $$ \boldsymbol{A}=\left[{\boldsymbol{\alpha}}_1\kern1em {\boldsymbol{\alpha}}_2\kern1em \dots \right] $$

It is helpful to distinguish two types of process units in modeling the steel system. A ‘real’ process is modeled by a *conversion process* with two output flows, a main product and a loss flow, parameterized by the efficiency η. For example, a blast furnace is modeled as a conversion process with two outputs, “pig iron” and “losses”. Various fractions of the pig iron are then sent to different steelmaking and casting processes; this is modeled by an *allocation process*, parameterized by a set of transfer coefficients. It is helpful to separate the conversion and allocation process steps, since the conversion process efficiencies are meaningfully related to the physical process, whereas the allocation is more arbitrary. These two process types correspond to two fundamental types of process identified by Pauliuk and colleagues (2016).

The column of transfer coefficients corresponding to a conversion process is

14 $$ {\boldsymbol{\alpha}}_j\left({\eta}_j\right)=\left[\begin{array}{c}\vdots \\ {}{\eta}_j\\ {}\vdots \\ {}1-{\eta}_j\\ {}\vdots \end{array}\right] $$

The locations of ${\eta}_j$ and $1-{\eta}_j$ in the vector correspond to the indices of the destination processes for the flows, with all other entries being 0. An allocation process produces a similar column vector ${\boldsymbol{\alpha}}_j\left({\boldsymbol{\varphi}}_j\right)$, where ${\boldsymbol{\varphi}}_j$ are the fractions of the output flow at process *j* allocated to each destination.

The model parameters therefore consist of external inflows ***q***, efficiency parameters $\boldsymbol{\eta} =\left\{{\eta}_j\right\}$ and allocation parameters $\boldsymbol{\varphi} =\left\{{\boldsymbol{\varphi}}_j\right\}$:

15 $$ \boldsymbol{\theta} =\left[\begin{array}{c}\boldsymbol{q}\\ {}\boldsymbol{\eta} \\ {}\boldsymbol{\varphi} \end{array}\right] $$

The model predictions consist of the flows, both external $\boldsymbol{q}=\left\{{q}_j\right\}$ and internal $\boldsymbol{z}=\left\{{z}_{jk}\right\}$:

16 $$ \boldsymbol{y}=\left[\begin{array}{c}\boldsymbol{q}\\ {}\boldsymbol{z}\end{array}\right] $$

which are defined in terms of the parameters ***θ*** by equations 10–14.

To model global steel flows, these conversion and allocation process units were assembled to replicate the model structure shown in the supporting information of Cullen and colleagues (2012). The resulting structure is detailed in the supporting information available on the Journal's website for this article.

### Observations of Model Predictions

Three types of data were available to inform the global steel model: flow rates between processes (e.g., production of pig iron from blast furnaces), external input flow rates (e.g., input of iron ore), and flows as fractions of total process throughputs (e.g., fraction of scrap input to steel casting). These data values were related to the model predictions by Gaussian errors, similar to equation 6:

17 $$ {\displaystyle \begin{array}{ccc}& & \left({d}_i|\boldsymbol{\theta} I\right)\hfill \\ {}& & \sim \left\{\begin{array}{cc}\mathcal{N}\left({z}_{JK},{\sigma}_i\right)\hfill & \mathrm{for}\kern4.pt \mathrm{observation}\kern4.pt \mathrm{of}\kern4.pt \mathrm{internal}\kern4.pt \mathrm{flow}\kern4.pt J\kern-0.16em \hspace{4.pt}\mathrm{to}\kern4.pt K\hfill \\ {}\mathcal{N}\left({q}_J,{\sigma}_i\right)\hfill & \mathrm{for}\kern4.pt \mathrm{observation}\kern4.pt \mathrm{of}\kern4.pt \mathrm{external}\kern4.pt \mathrm{inflow}\kern4.pt \mathrm{to}\kern4.pt \mathrm{process}\kern4.pt J\hfill \\ {}\mathcal{N}\left({z}_{JK}/{x}_K,{\sigma}_i\right)\hfill & \mathrm{for}\kern4.pt \mathrm{observation}\mathrm{s}\kern4.pt \mathrm{of}\kern4.pt \mathrm{input}\kern4.pt \mathrm{fraction}\kern4.pt \mathrm{of}\kern4.pt \mathrm{flow}\kern4.pt J\kern-0.16em \hspace{4.pt}\mathrm{to}\kern4.pt K\hfill \end{array}\right.\hfill \end{array}} $$

Observed data was added in two stages, using values quoted in the Supporting Information of Cullen and colleagues (2012). In the first stage, only data from a few readily‐available sources was used. After reviewing the resulting uncertain Sankey diagram, for the second stage additional sources describing the most uncertain areas were added. Details of the data used in each stage are given in the supporting information on the Web for this article. Note that the calculations performed by Cullen and colleagues to balance the analysis were not used, only the cited data values. For lack of better information, Gaussian uncertainty ranges of $\pm 10\%$ were applied to all values; in a real analysis it would be important to consider more carefully the confidence in the different observed data sources.

### Initial Knowledge about Model Parameters

Where original estimates of process yields are quoted in the Supporting Information of Cullen and colleagues (2012), these are taken as prior information for the process efficiency parameters ${\eta}_j$, using a logistic‐transformed normal distribution (details are given in the supporting information on the Web for this article). Only cited data values were included; process yields which were calculated by Cullen and colleagues to satisfy mass‐balance constraints were not used.

Most allocation parameters ${\boldsymbol{\varphi}}_j$ were given uniform Dirichlet prior distributions (see above), reflecting an initial lack of knowledge about allocations. However, Cullen and colleagues give estimates of the relative shares of casting and rolling losses which are lost, immediately reused, and reprocessed as scrap. To incorporate this information into the model, these parameters were assigned weak Dirichlet prior distributions centered on the estimated shares (details are given in the supporting information available on the Web for this article).

The remaining model parameters, the external inflow rates ***q***, were assigned uniform prior distributions.

### Software

The full data and code used to generate the Markov Chain Monte Carlo samples and to produce the figures in the article are available online (Lupton 2009) in the form of IPython Jupyter notebooks (Pérez and Granger 2007; Kluyver et al. 2009). They can be downloaded and run to reproduce the analysis, or viewed online.^2^

The code makes use of PyMC3 for doing the Markov Chain sampling (Salvatier et al. 2016), Matplotlib for plotting (Hunter 2007), and sankeyview and d3‐sankey‐diagram for creating the Sankey diagrams (Lupton 2009; Lupton and Allwood 2017).

### Results

The results of the Bayesian analysis are a set of samples calculated using Markov Chain Monte Carlo simulations. These can be visualized as Sankey diagrams showing the overall map of flows including uncertainty, as histograms of individual model parameters or flows, or as Sankey diagrams representing individual possible samples.

#### Uncertainties in Flows

**Figure 6:**
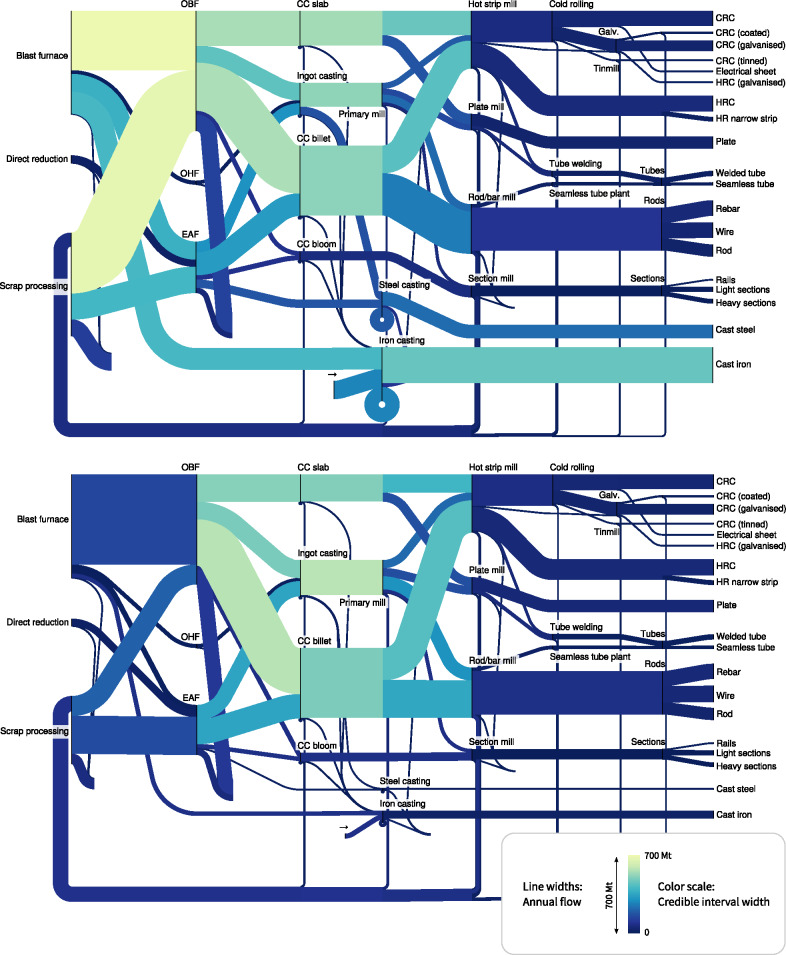
Sankey diagram showing uncertainty in flow values after stage 1 (top) and stage 2 (bottom) of the case study. The width of the lines corresponds to the mean parameter values. The shading indicates the width of the 95% credible interval, with darker colors showing more certain flows. The diagrams were produced from the posterior samples using sankeyview (Lupton and Allwood 2017). OBF = oxygen blown furnace; OHF = open hearth furnace; EAF = electric arc furnace; CC = continuous casting; CRC = cold rolled coil; HRC = hot rolled coil.

After the first stage, some flows are well defined, but considerable uncertainty remains in other areas. Figure 6 (top) shows a Sankey diagram representation of the results at this stage, with shading to show the uncertainty in the flow values. It clearly shows that while production of the semi‐finished products, on the right‐hand side, is well defined, considerable uncertainty remains about the upstream processes supplying these products. Production of cast steel and iron is also rather uncertain, because no data relating to these has been included yet.

In the second stage, additional data is added describing production of cast iron and steel, external sources of scrap, and the fraction of scrap used in casting, as well as the amount of pig iron used in electric arc furnaces (EAF). The result is shown in figure 6 (bottom). The uncertainty regarding the casting processes remains high, as no data on this part of the map has been supplied, but otherwise the flows are well defined. The next step would be to add additional data relating to these processes, but the results of this are not shown here.

#### Parameter Values

**Figure 7:**
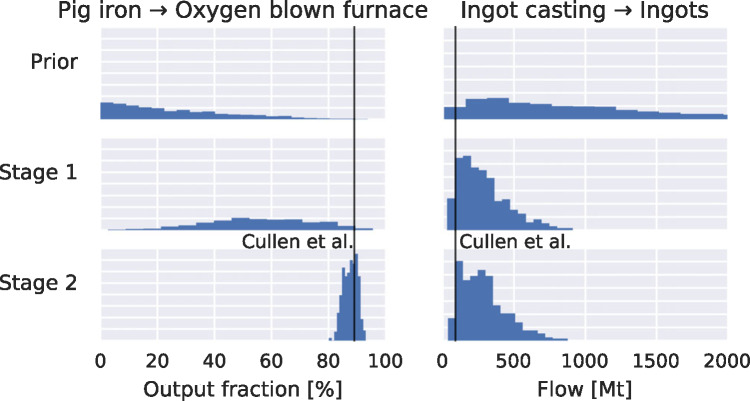
Increasingly precise knowledge about model parameters. Left: share of pig iron (blast furnace output) used in oxygen blown furnace (OBF). Initially the shares were completely unknown, with the mean share being 25% (out of 4 potential destinations). Stage 1 results favor a larger share for the OBF, while after stage 2, the share is known to be about 90% (coincident with Cullen et al.'s value, shown by the vertical line). Right: ingot casting production. Although the data reduces the initial uncertainty, a large range of possible values remain. The value found by Cullen and colleagues is within the region of high credibility.

As more information is progressively added, more is learned about the parameter values. Figure 7 shows histograms of a model parameter that represents the share of pig iron (the product of the blast furnace) that goes to the oxygen blown furnace (OBF). After stage 2, the value is quite well known. Production of ingots, also shown, remains more uncertain.

#### Individual Samples

Figure 6 shows the best‐estimate Sankey diagram, found from the mean value of the samples of the model parameters. Although this can be useful as a summary, it cannot show correlations; to get a full understanding of the possible alternative maps it is useful to view individual samples, especially when the variation is large (Tarantola 2005). An animation of possible Sankey diagrams is available online.^3^

## Discussion

This article has demonstrated that Bayesian inference is effective in allowing an incremental approach to MFA, making it easier for analysis to get started even in the face of missing and uncertain data, as well as providing a systematic method to track uncertainty, prioritize collection of new data, and incorporate new data as it becomes available.

In this article, it has been assumed that new data is already available in the form of a probability distribution. Usually this means providing an estimated standard deviation describing the uncertainty, but arbitrary probability distributions could also be directly specified. However, it is often unclear how the uncertainty should be chosen, based on what is known about the data source. Several methods have been proposed for data quality assessment and uncertainty characterization in MFA which seek to address this (Hedbrant and Sörme 2001; Laner et al. 2015; Schwab et al. 2016, 2017), which are complementary to the approach proposed here.

To start using this method, probability distributions must be assigned to represent initial knowledge about the model, and care should be taken in doing so to avoid unwanted biases. It was shown that previously‐used vague distributions for transfer coefficients include a bias towards equal allocation fractions, which is presumably unintended. The uniform Dirichlet distribution is a good alternative distribution.

As the analysis is developed, in many cases new information will lead to improved knowledge about the system, as demonstrated in the case study. But not every piece of new information will be helpful: some observations may be so uncertain they add little to what is already known, while others may contradict the current state of knowledge. In this case data reconciliation occurs naturally within the Bayesian framework.

### What Are the Disadvantages of This Approach?

A potential disadvantage of this approach is the computational cost of the Markov Chain Monte Carlo algorithms. The case study results took approximately 30 minutes to calculate on a 2 GHz Intel i5 laptop. Depending on the context, this may already be reasonable, and as little effort has yet been put into optimization, it seems likely that improvements can be made. Better understanding of the model structure can lead to more efficient sampling (Gelman et al. 2013), and some algorithms can be parallelized to take advantage of multi‐core processors (Goodman and Weare 2010).

The quantity of data required to perform an analysis in this way may seem to be greater than in other approaches, as prior distributions must be defined for every model parameter. In fact this is just a way of formalizing information which would be known and maybe taken into account anyway. By using formal prior distributions, the analysis is more transparent, as assumptions about model parameters are more clearly articulated.

### Future Work

Although Sankey diagrams are widely used to represent the results of material flow analyses, they are less commonly used to represent uncertainty. Graedel and colleagues (2004) used different dashed lines to represent confidence in their results, and Džubur and colleagues (2017) used color to present the “level of consistency” of different flows in a Sankey diagram. In this article uncertainty has been visualized using color and animation. The possibilities for visualizing uncertain MFA results should be explored further.

To keep the focus of this article on the Bayesian approach to iterative MFA, the case study presented above is based on a relatively simple, static MFA model. Beyond this, other types of MFA model, such as dynamic stock‐driven models (Müller 2006), may also be used within this overall approach. Dynamic models introduce new considerations, such as how the uncertain evolution of parameters over time should be treated, and there is significant scope for further investigation of the application of the Bayesian method to these and other MFA models.

Although this article has focused on identifying model parameters, Bayesian inference is also capable of doing model comparison. For example, Pauliuk and colleagues (2013) considered three different assumptions about the steel system, as different options in a sensitivity analysis. Rather than simply considering the range of outcomes stemming from these different assumptions, Bayesian inference provides a framework to quantify the probability of the different model assumptions, while intrinsically avoiding excessively complicated models which would over‐fit the data.

## Supplementary Information

**Supporting Information S1**: This supporting information gives more information about the material flow analysis (MFA) model used in the *Case Study: Global Steel Flows* section of the main article. Details are given for each of the three types of process from which the static model is built up: conversion processes, allocation processes, and sink processes.

## Footnotes

1 The probability of *X* is written as $P(X)$, while $P\left(X| YZ\right)$ is the conditional probability of *X* given that *Y* and *Z* are both true.

2 http://nbviewer.jupyter.org/github/ricklupton/bayesian‐mfa‐paper

3 https://doi.org/10.17863/CAM.8414
