# Supplementary material for: Incremental Material Flow Analysis with Bayesian Inference
Source: J Ind Ecol. 2017 Nov 27;22(6):1352–64. doi: 10.1111/jiec.12698 (PMC13091848; doi:10.1111/jiec.12698)
Supplement: Supplementary file 1 — Supporting Information S1: This supporting information gives more information about the material flow analysis (MFA) model used in the Case Study: Global Steel Flows section of the main article. Details are given for each of the three types of process from which the static model is built up: conversion processes, allocation processes, and sink processes. [file 44498_2018_2206007_MOESM1_ESM.pdf]

SUPPORTING INFORMATION FOR:

Lupton, R. C. and J. M. Allwood. 2017. Incremental material flow analysis with Bayesian inference. *Journal of Industrial Ecology.*

Summary

This supporting information gives more information about the material flow analysis (MFA) model used in the *Case Study: Global Steel Flows* section of the main article. Details are given for each of the three types of process from which the static model is built up: conversion processes, allocation processes, and sink processes.

1 Global steel model structure and prior distributions

This section gives more information about the material flow analysis (MFA) model used in the *Case Study: Global Steel Flows* section of the main article. The full data and code used to carry out the analysis and produce the figures in the article is available online (Lupton 2017) in the form of IPython Jupyter notebook (Pérez and Granger 2007; Thomas et al. 2016). They can be downloaded and run to reproduce the analysis, or viewed online[1].

The static MFA model is built up from three types of process: conversion processes, allocation processes, and sink processes. Details of each type of process are given in the following sections and tables.

1.1 Conversion processes

A 'real' process is modeled by a *conversion process* with two output flows, a main product and a loss flow, parameterized by the efficiency η (Equation (14) in the article). For example, a blast furnace is modeled as a conversion process with two outputs, "pig iron" and "losses". The conversion processes used in the model are listed in Table S1.

The connections between processes are defined by listing the destinations of the output flows from each process. The destinations of the product and loss outputs of the conversion processes are listed in Table S1, with the IDs corresponding to processes in Tables S1–S3.

The efficiencies η were modeled by logistic-transformed normal distributions in order to limit the value to the range $[0, 1]$:

$$\eta = \frac{1}{1 + e^{-x}} \tag{S1a}$$

$$x \sim \mathcal{N}(\mu, \sigma) \tag{S1b}$$

1. http://nbviewer.jupyter.org/github/ricklupton/bayesian-mfa-paper

Since in most cases estimates of the uncertainty of these efficiencies were not given by Cullen et al. (2012), a plausible range of values was chosen. The resulting mean values and the 95% confidence ranges are shown in the “prior” column of Table S1. In a few cases, independent estimates for efficiencies were not available, as the values given by Cullen et al. were the result of balancing the model. For these processes a wider uncertainty range was assigned.

1.2 Allocation processes

Allocation processes model the splitting of a flow to several destinations, parameterized by a set of transfer coefficients. For example, pig iron is sent to various steelmaking and casting processes in different proportions. The allocation processes in the model are listed in Table S2.

The destinations of the outputs of the allocation processes are listed in Table S2, with the IDs corresponding to processes in Tables S1–S3.

Most parameters were initially assigned uniform Dirichlet distributions, to reflect a lack of knowledge about the shares of products going to different destinations. In a few cases, to demonstrate that specific prior information about allocations can indeed be included if available, estimates of process losses’ destinations from Cullen et al. were included. These were modeled by concentrated Dirichlet distributions.

The Dirichlet probability density function is given by:

$$f(\boldsymbol{\phi}; \boldsymbol{\alpha}) = \frac{1}{B(\boldsymbol{\alpha})} \prod_{i=1}^{K} \phi_i^{\alpha_i - 1} \tag{S2}$$

where

$$B(\boldsymbol{\alpha}) = \frac{\prod_{i=1}^{K} \Gamma(\alpha_i)}{\Gamma\left(\sum_{i=1}^{K} \alpha_i\right)} \tag{S3}$$

in which Γ is the Gamma function and

$$\boldsymbol{\alpha} = \alpha_0 \boldsymbol{m} = (\alpha_1, \alpha_2, \ldots, \alpha_K) \tag{S4}$$

The mean vector $\boldsymbol{m}$ is normalized to sum to 1.

The allocation processes were parameterized by the concentration α_0 and the mean allocation vector $\boldsymbol{m}$. The results are listed in the “Dirichlet prior” columns of Table S2. The order of entries in $\boldsymbol{m}$ corresponds to the output IDs of each process listed in Table S2. The concentration factors α_0 were chosen to give a desired variance of one of the $\boldsymbol{\phi}$ by

$$\alpha_0 = \frac{m_i\,(1 - m_i)}{\mathrm{Var}(\phi_i) - 1} \tag{S5}$$

The variance of the other allocation coefficients $\boldsymbol{\phi}$ is determined by the Dirichlet distribution and cannot be set independently. Since in most cases estimates of uncertainty of allocation coefficients were not available, the target variance was chosen to give a plausible uncertain range of values.

1.3 Sink processes

The final set of processes are *sink processes*, which fall at the downstream end of the model. They simply act as placeholders for the outputs of other processes, and contribute no constraint equations to be model. The sink processes are listed in Table S3.

Table S1: Conversion processes and priors. Mean efficiency values are taken from Cullen et al. (numbers in square brackets refer to numbered flows in their Supplementary Information, which describes original sources). The choice of prior is discussed in the main text. CC = Continously cast, HR = Hot rolled, CR = Cold rolled.

ID	Name	Product destination	Loss destination	Prior for η (mean, 95% range)	Ref
BF	Blast furnace	PI	L	99.3% [98.4, 99.7]	[4]
DR	Direct reduction	DRI	L	99.3% [98.4, 99.7]	[1]
SP	Scrap preparation	S	L	85.0% [76.6, 90.8]	—
OBF	Oxygen blown furnace	OBFS	L	87.1% [81.8, 91.0]	[16]
OHF	Open hearth furnace	OHFS	L	87.1% [81.8, 91.0]	[20]
EAF	Electric arc furnace	EAFS	L	88.9% [80.2, 94.1]	[13]
CCBM	CC bloom	bloom	CCBML	95.5% [91.7, 97.6]	[28]
CCBT	CC billet	billet	CCBTL	97.5% [95.1, 98.8]	[34]
CCS	CC slab	slab	CCSL	96.5% [94.0, 98.0]	[39]
IC	Ingot casting	ingots	ICL	98.0% [96.6, 98.8]	[44]
PRM	Primary mill	PRMP	PRML	92.5% [91.4, 93.4]	[61]
IFC	Foundry iron casting	castiron	IFCL	66.0% [64.0, 68.0]	[56]
SPC	Steel product casting	caststeel	SPCL	52.2% [50.2, 54.2]	[52]
SEM	Section mill	SEMP	SEML	90.0% [87.8, 91.9]	[29]
RBM	Rod/bar mill	RBMP	RBML	94.0% [86.5, 97.5]	—
PLM	Plate mill	PLMP	PLML	90.0% [87.8, 91.9]	[73]
HSM	Hot strip mill	HSMP	HSML	96.0% [94.4, 97.2]	[78]
STP	Seamless tube plant	seamlesstube	SP	90.0% [64.4, 97.8]	—
TWP	Tube welding plant	weldedtube	SP	90.0% [72.0, 96.9]	—
CRM	Cold rolling mill	CRMP	SP	95.1% [94.0, 96.0]	[91]
GP-HR	Galvanizing plant (HR)	hrcgalv	SP	97.5% [96.9, 98.0]	[94]
GP-CR	Galvanizing plant (CR)	GPP-CR	SP	97.5% [96.9, 98.0]	[94]
TM	Tinmill	crctinned	SP	93.5% [93.0, 94.0]	[99]
OCP	Organic coating plant	crccoated	SP	98.0% [96.6, 98.8]	[102]

Table S2: Allocation processes and priors. Numbers in square brackets refer to flow numbers in the Supplementary Information of Cullen et al., which describes original sources. Where the allocation was not known, a uniform distribution was assigned. The Dirichlet parameters are described in the main text.

ID	Name	Destination IDs	Dirichlet prior		Ref
			α_0	$\boldsymbol{m}$	
PI	Pig iron	OBF, OHF, EAF, IFC	1.0	*uniform*	
DRI	Direct reduced iron	EAF	1.0	*uniform*	
S	Scrap	OBF, EAF	1.0	*uniform*	
OBFS	Oxygen blown furnace steel	CCS, CCBT, CCBM, IC	1.0	*uniform*	
OHFS	Open hearth furnace steel	IC	1.0	*uniform*	
EAFS	Electric arc furnace steel	CCBT, CCBM, IC, SPC	1.0	*uniform*	
bloom	Bloom	SEM	1.0	*uniform*	
billet	Billet	HSM, RBM	1.0	*uniform*	
slab	Slab	HSM, PLM	1.0	*uniform*	
ingots	Ingots	PRM, SPC	1.0	*uniform*	
PRMP	Primary mill products	SEM, RBM, PLM, HSM	1.0	*uniform*	
CCBML	CC bloom loss/scrap	L, SP, CCBM	19.6	[0.13, 0.16, 0.71]	[30-32]
CCBTL	CC billet loss/scrap	L, SP, CCBT	19.6	[0.13, 0.16, 0.71]	[36-38]
CCSL	CC slab loss/scrap	L, SP, CCS	19.6	[0.13, 0.16, 0.71]	[41-43]
ICL	Ingot casting loss/scrap	L, IC	3.7	[0.25, 0.75]	[47-48]
PRML	Primary mill loss/scrap	L, SP	3.0	[0.01, 0.99]	[62-63]
IFCL	Foundry iron casting loss/scrap	L, IFC	3.7	[0.25, 0.75]	[58]
SPCL	Steel product casting loss/scrap	L, SPC	3.7	[0.25, 0.75]	[53]
SEMP	Section mill products	secheavy, seclight, secrail	1.0	*uniform*	
RBMP	Rod/bar mill products	STP, rodrebar, rodwire, rodbar	1.0	*uniform*	
PLMP	Plate mill products	TWP, plate	1.0	*uniform*	
HSMP	Hot strip mill products	TWP, CRM, GP-HR, hrc, hrns	1.0	*uniform*	
SEML	Section mill loss/scrap	L, SP	4.9	[0.01, 0.99]	[67]
RBML	Rod/bar mill loss/scrap	L, SP	4.9	[0.01, 0.99]	[72]
PLML	Plate mill loss/scrap	L, SP	3.5	[0.01, 0.99]	[77]
HSML	Hot strip mill loss/scrap	L, SP	3.0	[0.01, 0.99]	[83]
CRMP	Cold rolling mill products	elsheet, crc, GP-CR, TM	1.0	*uniform*	
GPP-CR	Galvanizing (CR) products	crcgalv, OCP	1.0	*uniform*	

Table S3: Sink processes. CR = Cold rolled, HR = Hot rolled.

ID	Name
L	Loss
castiron	Cast iron
caststeel	Cast steel
crc	CR coil
crccoated	CR coil coated
crcgalv	CR coil galvanized
crctinned	CR coil tinned
elsheet	Electical sheet
hrc	HR coil
hrcgalv	HR coil galvanized
hrns	HR narrow strip
plate	Plate
rodbar	HR bar
rodrebar	Rebar
rodwire	Wire rod
seamlesstube	Seamless tube
secheavy	Sections (heavy)
seclight	Sections (light)
secrail	Sections (rail)
weldedtube	Welded tube

1.4 Observed data

To improve upon the prior information described in the tables above, observed data was added to the model in two stages, using values quoted in the Supplementary Information of Cullen et al. (2012).

In the first stage, only data from a few readily-available sources was used. These relate to flow values z_{jk} (see Equation (17) in the main article). For lack of better information, Gaussian uncertainty ranges of ±10% were applied to all values. The data are listed in Table S4, giving the source j and target k of the relevant flow value z_{jk}, the mean and standard deviation, and a reference to the original data source.

For the second stage additional sources describing the most uncertain areas were added. These include three further observations of flow values z_{jk}, one observation of an external input into the system q_j, and one value given as a fraction of the total destination process throughput (see Equation (17) in the main article). The second stage data is listed in Table S5.

The source and target process IDs in Tables S4 and S5 refer to the IDs listed in Tables S1–S3.

Table S4: Observed data added in stage 1. Numbers in square brackets refer to the Supplementary Information of Cullen et al. (2012), which describes the sources in more detail. worldsteel: worldsteel (2009a). SBB: Steel Business Briefing (2009).

Source	Target	Value [Mt]	Cullen ref	Data source
Flow values				
BF	PI	928.4 ± 50.0	[5]	worldsteel: Table 41
DR	DRI	65.8 ± 6.0	[2]	worldsteel: Table 42
EAF	EAFS	407.0 ± 40.0	[23]	worldsteel: Table 6
OBF	OBFS	891.7 ± 80.0	[24]	worldsteel: Table 6
OHF	OHFS	29.3 ± 3.0	[25]	worldsteel: Table 6
SEMP	seclight	44.4 ± 2.0	[105]	worldsteel: Table 16
SEMP	secheavy	39.6 ± 2.0	[106]	worldsteel: Table 15
SEMP	secrail	10.0 ± 1.0	[107]	worldsteel: Table 14
STP	seamlesstube	27.2 ± 2.0	[85]	worldsteel: Table 25
RBMP	rodrebar	147.0 ± 15.0	[108]	worldsteel: Table 17
RBMP	rodwire	148.5 ± 15.0	[109]	worldsteel: Table 19
RBMP	rodbar	109.7 ± 11.0	[110]	worldsteel: Table 18
TWP	weldedtube	44.5 ± 4.0	[89]	worldsteel: Table 26
PLMP	plate	110.0 ± 11.0	[111]	SBB
CRMP	elsheet	10.3 ± 2.0	[112]	worldsteel: Table 20
CRMP	crc	135.0 ± 13.0	[113]	SBB
GPP-CR	crcgalv	96.4 ± 9.0	[114]	worldsteel: Table 22
HSMP	hrc	150.0 ± 15.0	[115]	SBB
HSMP	hrns	40.0 ± 4.0	[116]	SBB
GP-HR	hrcgalv	10.0 ± 2.0	[97]	SBB
TM	crctinned	11.6 ± 2.0	[100]	worldsteel: Table 21
OCP	crccoated	16.5 ± 2.0	[103]	worldsteel: Table 23

Table S5: Observed data added in stage 2. Numbers in square brackets refer to the Supplementary Information of Cullen et al. (2012), which describes the sources in more detail.

Source	Target	Value	Units	Cullen ref	Data source
Flow values					
PI	EAF	44.6 ± 4.0	Mt	[12]	Barrington (2010)
SPC	caststeel	10.5 ± 2.0	Mt	[51]	Modern Casting (2009)
IFC	castiron	68.3 ± 6.0	Mt	[57]	Modern Casting (2009)
External inputs					
—	SP	475.5 ± 40.0	Mt	[10]	worldsteel (2009b)
Flows as fraction of destination					
S	IFC	0.17 ± 0.03	–	[54]	

References

Barrington, C. 2010. Ore Based Metallics: Overview of Global Trends (International Pig Iron Association).

Cullen, Jonathan M., Julian M. Allwood, and Margarita D. Bambach. 2012. Mapping the Global Flow of Steel: From Steelmaking to End-Use Goods. *Environmental Science & Technology* 46 (24): 13048–13055. doi:`10.1021/es302433p`.

Lupton, Richard C. 2017. Ricklupton/Bayesian-Mfa-Paper: Initial Published Version. *Zenodo.* doi:`10.5281/zenodo.581183`.

Modern Casting. 2009. *43rd Census of World Casting Production — 2008.*

Pérez, Fernando, and Brian E. Granger. 2007. IPython: A System for Interactive Scientific Computing. *Computing in Science & Engineering* 9 (3).

Steel Business Briefing. 2009. The World of Steel: Introduction to Steel, Module 3. London.

Thomas, Kluyver, Ragan-Kelley Benjamin, Pérez Fernando, Granger Brian, Bussonnier Matthias, Frederic Jonathan, Kelley Kyle, et al. 2016. Jupyter Notebooks – a Publishing Format for Reproducible Computational Workflows. In *Proceedings of the 20th International Conference on Electronic Publishing*, 87–90. doi:`10.3233/978-1-61499-649-1-87`.

worldsteel. 2009a. *Steel Statistical Yearbook 2009.* Brussels: World Steel Association.

———. 2009b. *World Steel in Figures 2009.* Brussels: World Steel Association.
